# Contrast-Enhanced Ultrasound in Distinguishing between Malignant and Benign Peripheral Pulmonary Consolidations: The Debated Utility of the Contrast Enhancement Arrival Time

**DOI:** 10.3390/diagnostics13040666

**Published:** 2023-02-10

**Authors:** Carla Maria Irene Quarato, Beatrice Feragalli, Donato Lacedonia, Gaetano Rea, Giulia Scioscia, Evaristo Maiello, Concetta Di Micco, Cristina Borelli, Antonio Mirijello, Paolo Graziano, Lucia Dimitri, Rosanna Villani, Marco Sperandeo

**Affiliations:** 1 Department of Medical and Surgical Sciences, Institute of Respiratory Diseases, Policlinico Universitario “Riuniti” di Foggia, University of Foggia, 71122 Foggia, Italy; 2 Department of Medical, Oral and Biotechnological Sciences, Radiology Unit, “G. D’Annunzio” University of Chieti-Pescara, 66100 Chieti, Italy; 3 Department of Radiology, “Vincenzo Monaldi” Hospital—AORN Ospedale Dei Colli, 80131 Naples, Italy; 4 Unit of Oncology, IRCCS Fondazione Casa Sollievo della Sofferenza, 71013 San Giovanni Rotondo, Italy; 5 Unit of Radiology, IRCCS Fondazione Casa Sollievo della Sofferenza, 71013 San Giovanni Rotondo, Italy; 6 Department of Internal of Medicine, IRCCS Fondazione Casa Sollievo della Sofferenza, 71013 San Giovanni Rotondo, Italy; 7 Unit of Pathology, IRCCS Fondazione Casa Sollievo della Sofferenza, 71013 San Giovanni Rotondo, Italy; 8 Department of Medical and Surgical Sciences, Institute of Internal Medicine, Liver Unit, Policlinico Universitario “Riuniti” di Foggia, University of Foggia, 71122 Foggia, Italy; 9 Unit of Interventional and Diagnostic Ultrasound of Internal Medicine, IRCCS Fondazione Casa Sollievo della Sofferenza, 71013 San Giovanni Rotondo, Italy

**Keywords:** contrast enhanced ultrasound, arrival time, lung ultrasound, peripheral pulmonary lesions, diagnostic accuracy

## Abstract

**Background.** Limited studies and observations conducted on a too small number of patients prevent determining the actual clinical utility of pulmonary contrast-enhanced ultrasound (CEUS). The aim of the present study was to examine the efficacy of contrast enhancement (CE) arrival time (AT) and other dynamic CEUS findings for differentiating between malignant and benign peripheral lung lesions. **Methods.** 317 inpatients and outpatients (215 men, 102 women; mean age: 52 years) with peripheral pulmonary lesions were included in the study and underwent pulmonary CEUS. Patients were examined in a sitting position after receiving an intravenous injection of 4.8 mL of sulfur hexafluoride microbubbles stabilized by a phospholipid shell as ultrasound contrast agent (SonoVue—Bracco; Milan, Italy). Each lesion was observed for at least 5 min in real-time and the following temporal characteristics of enhancement were detected: the arrival time (AT) of microbubbles in the target lesion; the enhancement pattern; the wash-out time (WOT) of microbubbles. Results were then compared in light of the definitive diagnosis of community acquired pneumonia (CAP) or malignancies, which was not known at the time of CEUS examination. All malignant cases were diagnosed by histological results, while pneumonia was diagnosed on the basis of clinical and radiological follow-up, laboratory findings and, in some cases, histology. **Results.** CE AT has not been shown to differ between benign and malignant peripheral pulmonary lesions. The overall diagnostic accuracy and sensibility of a CE AT cut-off value < 10 s in discriminating benign lesions were low (diagnostic accuracy: 47.6%; sensibility: 5.3%). Poor results were also obtained in the sub-analysis of small (mean diameter < 3 cm) and large (mean diameter > 3 cm) lesions. No differences were recorded in the type of CE pattern showed between benign and malignant peripheral pulmonary lesions. In benign lesions we observed a higher frequency of delayed CE wash-out time (WOT) > 300 s. Anyhow, a CE WOT cut-off value > 300 s showed low diagnostic accuracy (53.6%) and sensibility (16.5%) in discriminating between pneumonias and malignancies. Similar results were also obtained in the sub-analysis by lesion size. Squamous cell carcinomas showed a more delayed CE AT compared to other histopathology subtypes. However, such a difference was statistically significant with undifferentiated lung carcinomas. **Conclusions.** Due to an overlap of CEUS timings and patterns, dynamic CEUS parameters cannot effectively differentiate between benign and malignant peripheral pulmonary lesions. Chest CT remains the gold standard for lesion characterization and the eventual identification of other pneumonic non-subpleural localizations. Furthermore, in the case of malignancy, a chest CT is always needed for staging purposes.

## 1. Introduction

Despite the fact that peripheral pulmonary consolidations adherent to the pleura can be easily detected by ultrasound, the correlation between the ultrasound pattern and a specific pathology is quite poor [1,2,3]. Recently, contrast-enhanced ultrasound (CEUS) has been regarded as a valuable complementary ultrasound technique that can offer additional information to ultrasound gray-scale in B-mode [4].

Ultrasound contrast agents are composed of gas microbubbles stabilized in a phospholipid membrane, typically of about 6 µm in diameter (compared to a human erythrocyte measuring about 9 µm) [5]. Microbubbles are safer than iodinated- and gadolinium-based contrast material, particularly in patients with renal impairment [6]. Indeed, after several minutes in the circulation the microbubbles dissolve, the gas is exhaled from the lung and the phospholipid shell is metabolized mainly in the liver [5]. When exposed to the ultrasound beam, due to the compressibility of their gas cores, microbubbles vibrate about their equilibrium state and increase ultrasound backscatter by several orders of magnitude compared to a solid particle of the same size (i.e., erythrocytes), thus enhancing vascular contrast [7]. Unlike the contrast agents used in CT and MRI, ultrasound ones are purely intravascular contrast agents because microbubbles are too large to diffuse through the vascular endothelium into the interstitium. At the same time, however, they are small enough to cross thin capillary vessels that are below the detection threshold of power Doppler US because of a flow too slow to be differentiated from the surrounding tissue motion [4].

Given the advantage to allow a dynamic evaluation of tissue microvasculature and microperfusion in real time, CEUS has been successfully used in diagnostic imaging for a wide spectrum of pathological conditions. In Europe, CEUS use is currently approved for several cardiac and/or non-cardiac indications, including echocardiography, assessment of diseases in large vessels (such as aorta, carotid and intracranial vessels, peripheral arteries, renal arteries) and study of the microcirculation of parenchymatous organs (i.e., breast and focal liver lesion) [8]. Pulmonary CEUS applications, however, are still not licensed and are performed off-label.

Some authors assessed dynamic CEUS parameters and their capability to distinguish malignant from benign peripheral pulmonary consolidations [9]. In particular, the contrast enhancement (CE) arrival time (AT), that is the time taken for ultrasound contrast agent to reach the target lesion after injection, has been proposed as a useful tool to differentiate between benign lesions (that are mainly supplied by the pulmonary arterial system) and malignant lesions (that are mainly supplied by the systemic bronchial arterial system) [10,11,12]. The recommendations proposed by the European Federation of Societies for Ultrasound in Medicine and Biology (EFSUMB) stated that a time to enhancement of <10 s is indicative of a predominant supply from pulmonary arteries [8]. However, the same recommendations recognized that studies are limited and that the observations were conducted on too small a number of patients to allow a clear determination of the diagnostic value of CEUS in the evaluation of lung lesions [8,9].

On this background, the aim of the present study was to evaluate the diagnostic accuracy of CE AT in discriminating between malignant and benign peripheral pulmonary lesions, as recently described in the literature. Discriminatory accuracy of other dynamic CEUS findings was also assessed for completeness.

## 2. Materials and Methods

Between November 2021 and November 2022, we analyzed data of a total of 654 inpatients and outpatients scheduled for systematic TUS examination because of pulmonary consolidations on chest radiological imaging in our Unit of Interventional and Diagnostic Ultrasound of the Research Institute “Fondazione Casa Sollievo della Sofferenza Hospital” (San Giovanni Rotondo, Italy). All patients were in cardio-circulatory stability and showed no signs of respiratory distress.

The inclusion criteria were: (1) age > 18 years; (2) presence of subpleural pulmonary lesions defined as lesions not only abutting the pleura but also having an accessible ultrasound window; (3) availability of confirmation chest radiological images; (4) an informed written consent to perform a CEUS study. The exclusion criteria included the following: (1) known history of allergic reaction to the ultrasound contrast agent; (2) severe cardiovascular disease, including right-to-left shunts, severe pulmonary hypertension (i.e., pulmonary artery pressure > 90 mmHg) and uncontrolled systemic hypertension (i.e., systolic blood pressure > 140 mmHg); (3) pregnancy or breast-feeding; (4) massive pleural effusion (i.e., complete or near-complete opacification of the ipsilateral thorax on chest radiograph); (5) unsatisfactory ultrasound images; (6) failure to diagnose the lesion.

A total of 317 patients (215 men, 102 women; mean age: 52.12 ± 14.13 years) were finally included in the study. Their results were then compared in light of the definitive diagnosis of community acquired pneumonia (CAP) or malignancies, which was not known at the time of CEUS examination. Malignancies have been confirmed by biopsy and subsequent histological findings. CAP have been diagnosed on the basis of clinical and radiological follow-up, laboratory findings and, in some cases, histology.

The study followed the amended Declaration of Helsinki, the institutional Ethical Review Board of the hospital approved the protocol (TACE-CSS, n 106/2018) and all patients gave their informed consent to participate.

### 2.1. US and CEUS Examination

Ultrasound examination was independently performed by two physicians with over 20 years of experience in lung ultrasonography using an Esaote MyLab Twice scanner or, alternatively, an ESAOTE Technos MPX scanner (Genoa, Italy). For each exam we employed a multifrequency convex probe (3.5–5 MHz), the pre-setting for thoracic ultrasound in B-mode (i.e., gain compensation, 40–50%; dynamic range, 60–70 dB; depth, 70–140 mm; electronic imaging focus on the pleural line; tissue harmonics on) and US contrast setting (low mechanical index ≤ 0.1). Lungs were bilaterally explored, from the base to the apex, through all ventral, posterior and lateral intercostals spaces in order to identify consolidations. If more than one lesion was present, the largest and clearest one was selected. The maximal parallel and vertical diameters of the lesions were measured and the average diameter was recorded in cm.

Once the consolidation was clearly individuated on B-mode TUS scan, the US instrument was adjusted to a low-mechanical-index contrast enhancement mode. In all patients, US scans were performed in the sitting position in order to exclude a variability in contrast uptake due to changes in decubitus, potentially influencing the results.

Patients received then an intravenous injection of 4.8 mL of the new generation ultrasound contrast agent SonoVue (Bracco, Milan, Italy), followed by 10 mL of regular saline and the chronometer included in the scanner allowed the assessment of temporal characteristics of flow enhancement. The lesion was observed for at least 5 min in real-time in order to obtain maximum diagnostic information on lesion vascularity. The following temporal characteristics of enhancement were detected: the contrast enhancement arrival time (CE AT) between injection and appearance of microbubbles in the target lesion; the contrast enhancement (CE) pattern; the contrast enhancement wash-out time (CE WOT) between injection and disappearance of microbubbles. CE pattern was simply categorized using a dichotomous visual score, defining lesions as homogeneous or non-homogeneous, according to the distribution of more or less than 50% within the lesion. CE AT was classified as “early” if contrast agent reached the target lesion within 10 s. CE WOT was defined as “delayed” if the disappearance of contrast agent from the target lesion occurred after 300 s. CEUS images were recorded and stored as an average of 3 dynamic videoclips, each lasting about 2 min.

The clips were blindly reviewed by another operator with 35 years of experience. Cohen’s k values of the diagnostic results obtained for CE AT, CE pattern and CE WOT ranged from 0.81 to 1.00, indicating almost perfect agreement between operators.

### 2.2. Statistical Analysis

Data are presented as means ± standard deviation (SD) for continuous variables and as number (n) and percentage (%) for descriptive variables. Mann–Whitney U test was used for comparisons of continuous variables between patients with benign and malignant lesions and between patients with small (i.e., average diameter < 3 cm) and large lesions (i.e., average diameter ≥ 3 cm). Continuous variables between patients with different histopathology subtypes of malignant lesions were compared using Kruskal–Wallis test. Dunn’s multiple comparison test was performed to determine whether there was a difference between the mean rank of all the possible pairs.

Pearson’s chi-squared test was used to assess statistically significant difference in descriptive variables between patients with benign and malignant lesions, patients with small and large lesions and patients with different histopathology subtypes of malignant lesions. Significance was established at a *p*-value < 0.05. Dynamic CEUS parameters diagnostic accuracy, sensitivity, specificity, positive and negative predicted values were calculated with a 95% confidence interval (CI).

## 3. Results

### 3.1. Participant Characteristics

Among the 317 participants, 170 patients were finally diagnosed with benign consolidations and 147 with malignant lesions. The two groups did not differ significantly in term of sex (*p* = 0.91) or smoking habit (*p* = 0.57). However, patients with benign consolidations were younger than those with malignancies (*p* < 0.0001). The average diameter of lesions measured on US was 3.2 ± 0.9 cm for community acquired pneumonia and 2.7 ± 0.5 cm for malignant lesions; malignancies were significantly smaller than benign consolidations (*p* = 0.0004). A mild to moderate pleural effusion was mostly associated to malignant lesions, despite with no statistically significant difference (*p* = 0.31) (Table 1).

All 147 malignant cases were diagnosed by biopsy. Histological diagnoses of malignancies are shown in Table 2. Of the 170 benign cases, 134 (78.8%) were diagnosed based on clinical course, imaging follow-up and laboratory results, while 36 (21.2%) had also clear histology results.

### 3.2. Comparison of CEUS Parameters between Benign and Malignant Lesions

No evidence for differences in the CE AT was found between benign and malignant lesions (26.8 ± 7.4 vs. 27.0 ± 6.5; *p* = 0.39). No differences were recorded in the frequencies of lesions showing and “early” CE AT between the two groups (5.29% vs. 3.40%; *p* = 0.67). No differences were recorded in the number of lesions that showed an homogeneous CE pattern (76.5% vs. 72.8%) or a non-homogeneous CE pattern (23.5% vs. 27.2%) between the two groups (*p* = 0.52). Although without reaching statistical significance, an homogeneous CE pattern was the most frequent presenting feature compared to the non-homogeneous CE pattern for both benign (76.5% vs. 23.5%) and malignant (72.8% vs. 27.2%) lesions (*p* = 0.52). Malignant lesions showed statistical significance for an earlier CE WOT compared to benign lesions (249.7 ± 18.1 vs. 281.4 ± 22.7; *p* < 0.0001). In the group of benign lesions we observed a statistically higher frequency of consolidations showing a delayed CE WOT (16.47% vs. 3.40%; *p* = 0.0001) (Table 3).

**Figure 1:**
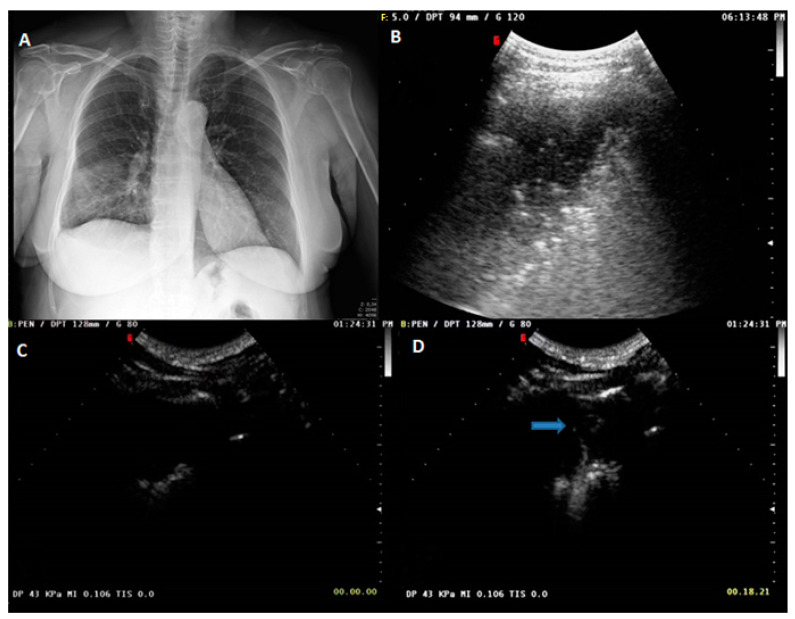
(**A**) Chest radiograph showing ill-defined patchy consolidation and ground glass opacities (GGO) in the right middle to lower lung zone. The clinical–radiological diagnosis was pneumonia. (**B**) US scan (thoracic pre-setting) in B-mode showing a hypoechoic subpleural pulmonary lesion with inner hyperechoic spots. (**C**) CEUS scan with SonoVue at 0 s showing no lesion perfusion. (**D**) CEUS scan with SonoVue at 18 s showing first spots of arrival of the ultrasound contrast agent (blue arrow).

A CE AT cut-off of <10 s showed an overall diagnostic accuracy of 47.6% (95% CI: 42.0–53.3%), a sensitivity of 5.3% (95% CI: 2.5–9.8%), a specificity of 96.6% (95% CI: 92.2–98.9%), a positive predictive value of 64.3% (95% CI: 38.2–84.0%) and a negative predictive value of 46.9% (95% CI: 45.7–48.0%) in discriminating benign from malignant lesions (Figure 1). A CE WOT cut-off of >300 s showed an overall diagnostic accuracy of 53.6% (95% CI: 48.0–59.2%), a sensitivity of 16.5% (95% CI: 11.2–22.9%), a specificity of 96.6% (95% CI: 92.2–98.9%), a positive predictive value of 84.9% (95% CI: 68.9–93.4%) and a negative predictive value of 50.0% (95% CI: 48.0–59.2%) in discriminating CAP from malignancies.

### 3.3. CEUS Parameters between Lesions of Different Sizes

Depending on the average diameter, the participants were divided into two groups: the small lesion group (average diameter < 3 cm) and the large lesion group (average diameter ≥ 3 cm).

No differences were recorded in the CE AT between benign and malignant lesions for both the small lesion group (25.0 ± 6.6 vs. 25.7 ± 6.3; *p* = 0.46) and the large lesion group (27.7 ± 6.5 vs. 28.4 ± 7.9; *p* = 0.92). Lesions in the small lesion group showed earlier arrival times than lesions in the large lesion group. However, while this difference showed statistical significance for benign lesions (25.0 ± 6.6 vs. 28.4 ± 7.9; *p* = 0.04), the same was not observed for malignant lesions (25.7 ± 6.3 vs. 27.7 ± 6.5; *p* = 0.07). No differences were recorded in the number of lesions that showed an homogeneous CE pattern (78.8% vs. 69.8; *p* = 0.23 and 74.4% vs. 78.4%; *p* = 0.68) or a non-homogeneous CE pattern (21.2% vs. 30.2%; *p* = 0.23 and 25.6% vs. 21.6%; *p* = 0.68) between benign and malignant lesions in the two groups. Benign lesions continued to show statistical significance for a delayed CE WOT compared to malignant lesions in both the small lesion group (250.8 ± 15.7 vs. 282.1 ± 25.4; *p* < 0.0001) and the large lesion group (247.5 ± 22.3 vs. 280.7 ± 20.1; *p* < 0.0001) (Table 4).

In the small lesion group, a CE cut-off of <10 s showed a diagnostic accuracy of 55.7% (95% CI: 48.0–63.2%), a sensitivity of 6.3% (95% CI: 2.0–14.0%), a specificity of 96.9% (95% CI: 91.1–99.4%), a positive predictive value of 62.5% (95% CI: 29.1–87.1%) and a negative predictive value of 55.4% (95% CI: 53.7–57.0%). In the large lesion group, a CE cut-off of <10 s showed a diagnostic accuracy of 37.6% (95% CI: 29.6–46.1%), a sensitivity of 4.4% (95% CI: 1.2–11.0%), a specificity of 96.1% (95% CI: 86.5–99.5%), a positive predictive value of 66.7% (95% CI: 27.5–91.3%) and a negative predictive value of 36.3% (95% CI: 34.7–38.0%). In the small lesion group, a CE WOT cut-off of >300 s showed an overall diagnostic accuracy of 60.8% (95% CI: 53.2–68.1%), a sensitivity of 17.5% (95% CI: 9.9–27.6%), a specificity of 97.0% (95% CI: 91.1–99.4%), a positive predictive value of 82.4% (95% CI: 58.2–94.0%) and a negative predictive value of 58.5% (95% CI: 55.9–61.1%). In the large lesion group, a CE WOT cut-off of >300 s showed an overall diagnostic accuracy of 44.7% (95% CI: 36.3–53.3%), a sensitivity of 15.6% (95% CI: 8.8–24.7%), a specificity of 96.1% (95% CI: 86.5–99.5%), a positive predictive value of 87.5% (95% CI: 62.4–96.7%) and a negative predictive value of 39.2% (95% CI: 36.7–41.7%).

### 3.4. US and CEUS Parameters in Different Histopathology Subtypes of Malignant Lesions

US and CEUS characteristics in the different histopathology subtypes of malignant subpleural lesions are detailed in Table 5.

**Figure 2:**
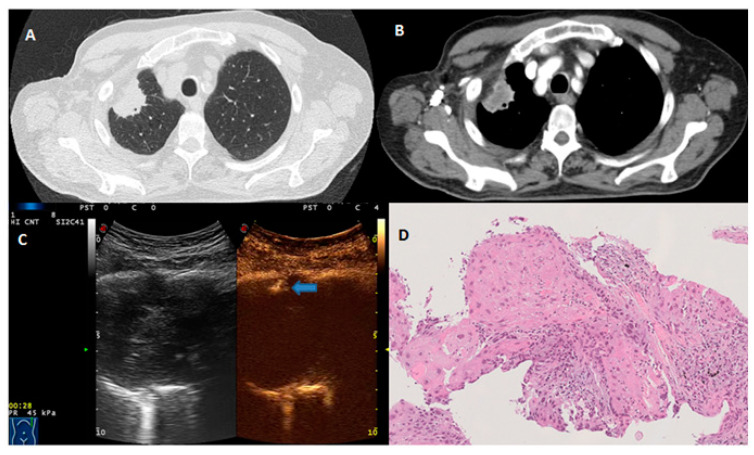
(**A**) Axial chest CT scan (lung window) showing a subpleural pulmonary lesion in the upper lobe of the right lung. (**B**) Axial contrast-enhanced CT scan (mediastinal window) showing inner necrosis in the same subpleural pulmonary lesion. (**C**) CEUS scan with SonoVue showing the arrival time of the ultrasound contrast agent (blue arrow) at 28 s. (**D**) Histopathological examination showing multiple keratinizing foci (hematoxylin and eosin ×10). The final diagnosis was squamous cell lung carcinoma.

**Figure 3:**
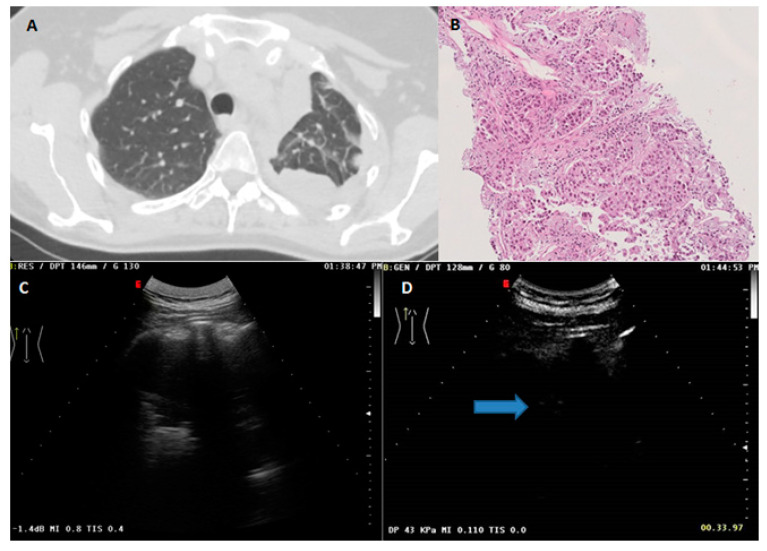
(**A**) Axial chest CT scan (lung window) showing a subpleural pulmonary lesion of the upper lobe extending in the superior segment of the lower lobe in the left lung. (**B**) Histopathological examination showing a predominant acinar pattern (hematoxylin and eosin ×10). The final diagnosis was lung adenocarcinoma. (**C**) US scan (thoracic pre-setting) in B-mode showing a mixed hyper- hypoechoic subpleural pulmonary lesion. (**D**) CEUS scan with SonoVue showing first spots of arrival of the ultrasound contrast agent (blue arrow) at 33 s.

No evidence for statistically significant differences in terms of average diameter of lesions measured on US was assessed between different histopathology subtypes of malignant lesions (*p* = 0.35). Squamous cell carcinoma showed a more delayed CE AT compared to other histopathology subtypes (*p* = 0.35). However, Dunn’s multiple comparison test assessed the existence of a statistically significant difference only between squamous cell carcinomas and undifferentiated lung carcinomas (30.7 ± 8.1 vs. 24.4 ± 5.7; *p* = 0.004) (Figure 2 and Figure 3). No differences were recorded in the frequencies of lesions with an homogeneous CE pattern or a non-homogeneous CE pattern between different histopathology subtypes (*p* = 0.75). No evidence for statistically significant differences in the CE WOT was found between different malignant histotypes (*p* = 0.24).

## 4. Discussion

Results of studies assessing CE AT values for the characterization of peripheral pulmonary lesions are controversial. While some authors have observed that CE AT allows to effectively distinguish between benign and malignant subpleural pulmonary lesions [10,11,12], others have reported inconclusive results [13,14]. Furthermore, cut-off values of CE AT identified by studies reporting positive results are variable. Caremani et al. [10] proposed an AT greater than 10 s for identification of malignant lesions, while Sartori et al. [11] suggested a cut-off of 7.5 s. The study by Bi et al. [12] confirmed that both cut-off values can be good discriminators in peripheral lung lesions, but slightly lower diagnostic results have been observed in smaller lesions (vertical diameter < 3 cm). As noted in a systematic review by Jacobsen et al. [9], an appropriate comparison by the various studies in the literature could not be made because of the limited number of available studies and the heterogeneity in the assessed dynamic CEUS parameters. Indeed, some authors defined and employed other CEUS diagnostic criteria, such as the lesion-lung CE AT difference (i.e., the CE AT difference between lesion and air-filled lung tissue) [15,16] and the CE AT difference ratio (i.e., the ratio of CE AT difference between lesion and air-filled lung tissue to CE AT difference between thoracic wall and air-filled lung tissue) [12], which they claimed to be superior to the classical CE AT of lesion alone in differentiating between malignancies and benign processes.

To date, a CE AT cut-off of 10 s has been included in the recommendations for the clinical practice of CEUS in non-hepatic applications proposed by the EFSUMB [8]. Results of our study, however, showed that such timing of arrival of CE in the target lesion did not allow to significantly distinguish between pneumonias and malignancies.

The lung is characterized by dual a blood circulation: the pulmonary arterial system and the bronchial arterial system. The pulmonary arterial system carries systemic deoxygenated blood from the right ventricle to the lungs. Branches of the pulmonary artery travel closely alongside the bronchial tree on their way to the alveoli. Here, the blood passes through a nest of small capillaries and becomes oxygenated. Oxygenated blood from the lungs is then circulated back to the heart through the pulmonary veins that drain into the left atrium [17]. The bronchial arterial system arises from the descending aorta (and/or from the proximal first intercostal artery with considerable anatomic variation) and carries oxygenated blood from the left ventricle to the entire bronchial tree itself (with the exception of the alveoli), the interstitial tissue, pulmonary vessels and the visceral pleura. The venous deoxygenated blood of the bronchial circulation drains in part into the veins of the systemic circle that go to the right heart and, in a lesser part, into the pulmonary veins that run into the left heart, as a component of the physiological right–left shunt [17].

The inflammatory process that characterizes pneumonia mainly affects the terminal alveoli and bronchioles, leading to the ectasia and permeabilization of the nest of the blood capillaries by which each alveolus is surrounded and that are supplied by small branches of the pulmonary artery. This explanation was given to justify the early CE AT that would characterize inflammatory lesions on CEUS study [10]. On the other hand, a delayed CE AT in lung malignancies was justified with the uncontrolled proliferative phenomena and the neoangiogenesis processes that occur in the tumor microenvironment. Neoplastic cells grow uncontrollably until they find themselves in conditions of oxygen deficiency due to the increase in the size of the tumor mass [18]. Pulmonary arteries seem to have no or very low capacity for neoangiogenesis. Hence, the proliferating vasculature and blood supply from the bronchial arteries markedly increases and gradually replaces the supply from the pulmonary artery along with the growth of the tumor mass [19].

**Figure 4:**
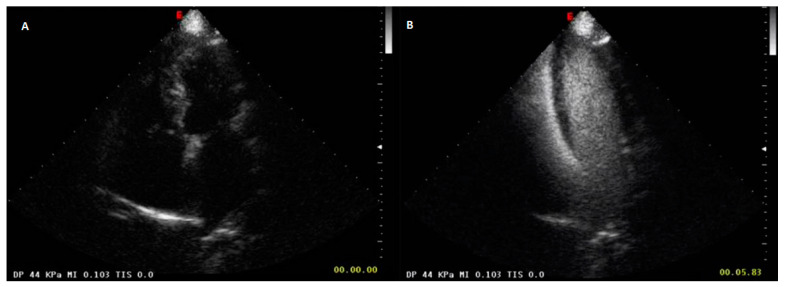
(**A**) US scan showing the four cardiac chambers before administration of contrast medium in a normal subject. (**B**) CEUS scan with SonoVue at 6 s after administration of contrast medium. All the four cardiac chambers are filled with contrast and therefore the double pulmonary circulation is completely perfused.

Anyhow, previous studies on contrast-enhanced magnetic resonance (MR) have shown that in healthy individuals contrast enhancement appears in the right heart between 1 and 4.5 s after injection (indicating the time window of pulmonary arterial vascularity), and between 6 and 7.5 s in the left heart [20,21]. As a result, in the normal lung, after only 6–7.5 s from the infusion of the contrast, the four cardiac chambers will be completely perfused (i.e., the double circulation is almost completed) (Figure 4). Therefore, the ecocontrastographic evaluation of lung subpleural lesions reveals only the presence of vascularization but does not allow to differentiate with confidence the pulmonary artery blood supply from the bronchial one, except for those rare cases in which enhancement is evident before 6 s.

In our study, none of the benign nor malignant lesions showed enhancement before 6 s. Furthermore, a CE AT ≤ 10 s was observed in nine cases (5.29%) of benign lesions and in 5 cases (3.40%) of malignant lesions, with no statistically significant difference (*p* = 0.67). A CE AT cut-off value ≤ 10 resulted in a diagnostic accuracy of 47.6% and a sensibility of only 5.3% in discriminating pneumonias from malignancies.

Indeed, it is important to remember that the permeabilization of the capillary network in pneumonia is followed by alveolar congestion with fibrino-purulent and fibrino-haematic material and the consequent creation of areas of ventilation/perfusion ratio mismatch. The pulmonary circulation system has the peculiarity to create vasoconstriction when confronted with local hypoxia, while systemic arteries will dilate in order to improve tissue perfusion. Therefore, vasoconstriction will occur in poorly ventilated regions of the lung in order to redirect blood flow to better-ventilated regions [17]. Furthermore, it is noteworthy that bronchial vessels supply the intrapulmonary airways at about the level of the terminal bronchioles where they form extensive anastomoses with the pulmonary vasculature. Anastomoses between the two systems are usually closed in healthy lungs. However, in case of hypoxia the anastomoses will be opened and nutrition of this region will be done via bronchial arteries [17]. In addition, a disorganized and chaotic vascular pattern can be seen also in tissue samples of chronic inflammatory processes as evidence of bronchial artery neoangiogenesis [22]. To this regard, in the study by Hong-Xia et al. [15], CE AT was significantly shorter in pneumonia compared to malignant tumors or chronic inflammation, whereas no difference was seen between malignant tumors and chronic inflammatory pseudotumors.

In our study, CE AT did not allow to distinguish between CAP and neoplasms even in the subgroup analysis of lesions of different sizes. The small-lesion group showed shorter CE AT than the large-lesion group for both benign and malignant lesions. This might be explained by a greater percentage of blood supply from the pulmonary circulation in the small-lesion group and by the presence of a greater amount of hypoxia and subsequently increased opening of the shunts with the bronchial circulation in the large-lesion group. Anyhow, the sensibility of a CE AT cut-off value ≤ 10 in distinguishing benign lesions from malignant ones was of only 6.3% in the small-lesion group and only 4.4% in the large-lesion group.

Regarding other dynamic CEUS findings, the CE pattern did not differ significantly between benign peripheral pulmonary lesions and malignant ones (*p* = 0.52). A homogeneous distribution of CE was found in 130/170 (76.5%) pneumonias and in 107/147 (72.8%) malignancies, whereas a non-homogeneous distribution was observed in 40/147 (23.5%) CAP lesions and 40/147 (27.2%) neoplasms. Probably, the lack of significant differences of CEUS features between CAP and malignancies could reflect some pathophysiological aspects shared by these conditions. For example, also local vasoconstriction due to hypoxic stimuli is usually present within the heterogeneous microenvironment of benign inflammatory processes and malignant lesions may be imaged as a defect in US contrast enhancement [17]. Additionally, the compressive effect of a moderate effusion could generate distortion in blood flow that may influence CE distribution. Although with no statistically significant difference, in our experience a mild to moderate pleural effusion mostly accompanies malignant lesions. Moreover, in complicated pneumonias, foci of hypoenhancement may correspond to areas of inflammatory cell infiltration, fibroblast proliferation and interstitial fibrous tissue [22]. Even areas with no opacification can be seen, indicating the presence of lung abscesses, necrosis or hemorrhage [23].

Finally, in the present experience, malignant lesions exhibited more rapid contrast wash-out times compared to pneumonias. This could be due to the presence of immature irregular and tortuous vessels and a large number of abnormal arterio-venous shunts associated with neoangiogenesis in malignancies. On the other hand, the delayed enhancement in pneumonias was likely due to a widespread vasoconstriction caused by hypoxia, unaccompanied or accompanied by much more minimal phenomena of neoangiogenesis [10,11]. However, even a CE WOT cut-off >300 did not prove to be an effective parameter in discriminating benign from malignant lesions, showing an overall diagnostic accuracy of 53.6% and a sensitivity of only 16.5%.

Considering the various types of lung malignances, CE parameters may be very heterogeneous. In this study, squamous carcinomas showed later enhancement arrival times compared to other histotypes. However, the existence of a statistically significant difference was demonstrated only between squamous cell carcinomas and undifferentiated lung carcinomas. Therefore, the variable times to enhancement might be related to the histological differentiation degree of malignancy and the rate of neovascularization. On the other hand, observed CE patterns did not allow the various histotypes of lung cancer or metastases to be distinguished from each other.

That said, the lesion microenvironment could only partly account for the indiscriminate information provided by CEUS in CAP and malignancies. A lot of physiological and pathological conditions, such as sitting or supine position of the patient, hyperthyroidism and hypothyroidism, tachycardia or bradycardia, chronic heart failure or chronic pulmonary disease implying local circulatory shunt and many other confounders can modify the standard CE transit time in the lungs [24]. The strengths of our study mainly consist of the inclusion of a large number of unselected inpatients and outpatients in a single center and in having examined all patients in a sitting position in order to avoid bias related to the redistribution of the circulation when passing from a sitting to supine position. Anyhow, although in this study we have included a relatively high number of younger patients with CAP compared to those with lung malignancies, we have not assessed the presence comorbidities that could also have influenced our results. Patients with different pathological types of lesions and different concomitant pulmonary and cardiovascular diseases will have to be included in future studies to clarify the effect of these factors.

Another important limitation relies in the fact that US and even more CEUS are strictly operator-dependent techniques. Although we estimated a good interobserver agreement on recorded clips, this may not reflect the daily routine practice of CEUS, usually performed by several operators. Furthermore, currently, there is still a lack of studies exploring the curriculum of basic skills needed in CEUS through learning curves.

**Figure 5:**
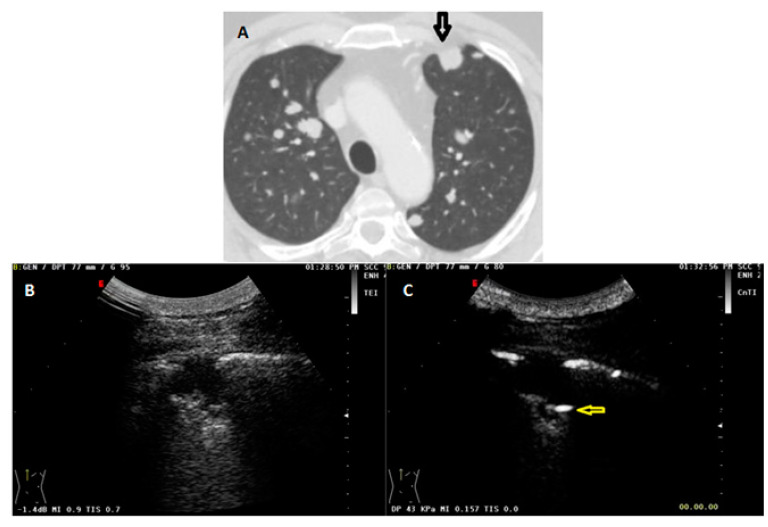
(**A**) Axial chest CT scan (lung window) showing a subpleural malignant pulmonary lesion in the anterior segment of the left upper lobe (black arrow). (**B**) The corresponding US B-mode scan showed a subpleural hypoechoic lesion with a hyperechoic spot within. (**C**) In the US scan with contrast setting (mechanical index: 0.157), but without the administration of contrast agent, seems to be even enhanced (yellow arrow).

**Figure 6:**
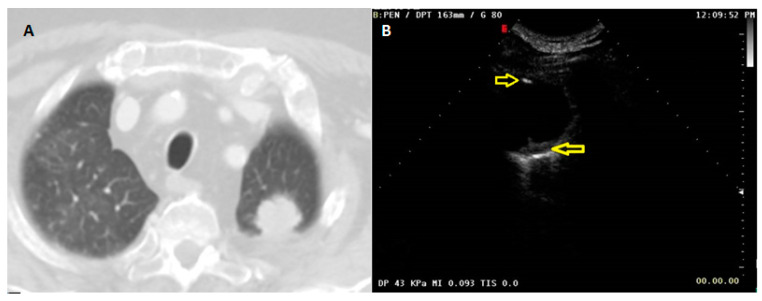
(**A**) Axial chest CT scan (lung window) showing a subpleural malignant pulmonary lesion in the apicoposterior segment of the left upper lobe. (**B**) US scan with contrast setting (mechanical index: 0.093), but without the administration of contrast agent, displaying hyperechogenic spots inside the lesion (yellow arrows) which could be confused with CE AT.

**Figure 7:**
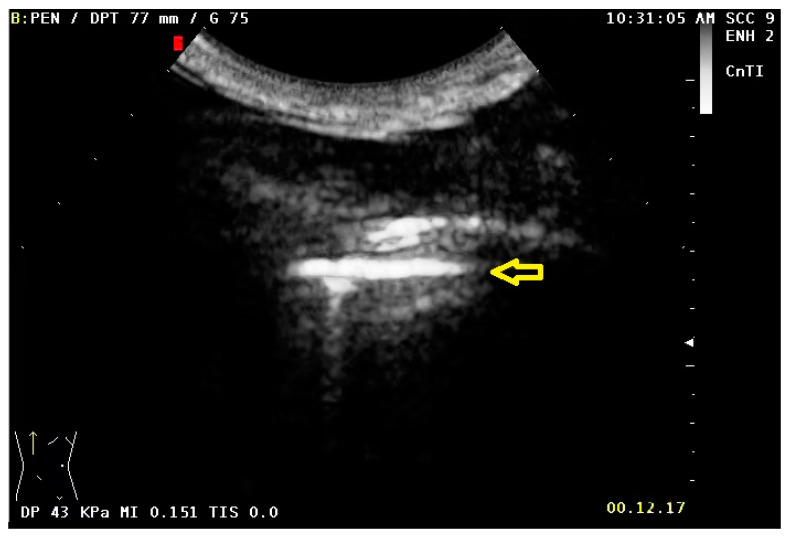
Duplication of the hyperechoic pleural line 10 s after administration of the US contrast agent (yellow arrow).

Finally, as with B-mode US, image artifacts could be encountered when performing CEUS examination that must be recognized to improve interpretation of CEUS findings [25]. For example, any spots within the lesion that are hyperechoic before contrast injection can be visually misclassified as first spots of enhancement due to the arrival of contrast in the target lesion (Figure 5 and Figure 6). The same can be said for reverberation artefacts which may be produced below the pleural line upon arrival of the contrast (Figure 7).

These CEUS artifacts could clearly be responsible for misinterpretation relating, above all, the use of other suggested CEUS diagnostic criteria, such as the lesion-lung CE AT difference and the CE AT difference ratio.

## 5. Conclusions

In conclusion, our results show that diagnostic criteria determined from contrast-enhanced US cannot effectively differentiate the blood supply of peripheral pulmonary lesions.

Due to an overlap of CEUS timing and patterns between peripheral benign inflammatory and malignant pulmonary lesions, additional radiological studies (such as chest radiograph, CT and, in case of further doubts, a PET-CT) are strongly warranted. Anyway, in suspicious cases a chest CT remains necessary for staging purposes, while the histological examination of the lesions is always needed to confirm the diagnosis and to obtain immunohistochemical characterization. To this regard, US contrast media could be used to improve the diagnostic yield of percutaneous biopsy. In pneumonia cases, the possible addition of a simple ultrasound examination in B-mode could help to visualize the regression of the consolidation (if the lesion is adherent to the pleural surface visible on US). Anyhow, a CT must still be done to exclude the possibility of further lesions.

## Figures and Tables

**Table 1:** Demographical and clinical characteristics of the patients in the study.

Variable	Benign (n = 170)	Malignant (n = 147)	*p*-Value
Age, years; mean ± SD (min-max)	40.4 ± 9.1 (19–81)	65.7 ± 5.7 (50–88)	<0.0001
Sex:			
Male; n, %	102 (60.0%)	86 (58.5%)	0.91
Female; n, %	68 (40.0%)	61 (41.5%)	
Smokers; n, %	104 (61.2%)	85 (57.8%)	0.57
Lesion diameter, cm	3.2 ± 0.9 (1.5–8.0)	2.7 ± 0.5 (1.25–5.75)	0.0004
Pleural effusion; n, %	84 (49.4%)	81 (55.1%)	0.31

**Table 2:** Malignancies histopathology subtypes.

Histopathology Subtype	Malignancies (n = 147)
Lung metastasis	12 (8.2%)
Small cell lung cancer	7 (4.8%)
Squamous cell lung carcinoma	35 (23.8%)
Adenocarcinoma	60 (40.8%)
Undifferentiated lung carcinoma	33 (22.4%)

**Table 3:** Comparison of CEUS parameters between benign and malignant lesions.

Overall Lesions	Benign (n = 170)	Malign (n = 147)	*p*-Value
CE Arrival Time (AT)	26.8 ± 7.4	27.0 ± 6.5	0.39
Early CE AT ≤ 10 s	9 (5.29%)	5 (3.40%)	0.67
Homogeneous CE	130 (76.5%)	107 (72.8%)	0.52
Non-homogeneous CE	40 (23.5%)	40 (27.2%)	
Wash-out Time (WOT)	281.4 ± 22.7	249.7 ± 18.1	<0.0001
Delayed CE WOT > 300 s	28 (16.47%)	5 (3.40%)	0.0001

**Table 4:** Comparison of CEUS parameters between benign and malignant lesions in the small lesion group (average diameter < 3 cm) and the large lesion group (average diameter ≥ 3 cm).

CEUS Parameters	<3 cm (n = 176)			≥3 cm (n = 141)		
	Benign (n = 80)	Malign (n = 96)	*p*-Value	Benign (n = 90)	Malign (n = 51)	*p*-Value
Arrival Time (AT)	25.0 ± 6.6	25.7 ± 6.3	0.46	28.4 ± 7.9	27.7 ± 6.5	0.92
Early CE AT ≤ 10 s	5 (6.3%)	3 (3.1%)	0.32	4 (4.4%)	2 (3.9%)	0.88
Homogeneous CE	63 (78.8%)	67 (69.8%)	0.23	67 (74.4%)	40 (78.4%)	0.68
Non-homogeneous CE	17 (21.2%)	29 (30.2%)		23 (25.6%)	11 (21.6%)	
Wash-out Time (WOT)	282.1 ± 25.4	250.8 ± 15.7	<0.0001	280.7 ± 20.1	247.5 ± 22.3	<0.0001
Delayed CE WOT > 300 s	14 (17.5%)	3 (3.1%)	0.001	14 (15.6%)	2 (3.9%)	0.08

**Table 5:** Comparison of CEUS parameters between different histopathology subtypes of malignant lesions (LM, lung metastasis; PM, pneumonia; R-CAP, refractory to standard treatment community-acquired pneumonia; SCLC, small cell lung cancer; SCC, squamous cell lung carcinoma; ADLC adenocarcinoma lung cancer; UDLC, undifferentiated lung carcinoma).

US/CEUS Parameters	LM (n = 12)	SCLC (n = 7)	SCC (n = 35)	ADLC (n = 60)	UDLC (n = 33)	*p*-Value
Lesion diameter, cm	2.9 ± 0.6	2.7 ± 0.6	2.6 ± 0.5	2.8 ± 0.5	2.7 ± 0.4	0.35
Arrival Time	24.1 ± 4.2	25.4 ± 3.6	30.7 ± 8.1	27.0 ± 6.4	24.4 ± 5.7	0.03
Homogeneous CE	7 (58.3%)	6 (85.7%)	26 (74.3%)	44 (73.3%)	24 (72.7%)	0.75
Non-homogeneous CE	5 (41.7%)	1 (14.3%)	9 (25.7%)	16 (26.7%)	9 (27.3%)	
Wash-out Time	242.1 ± 14.9	251.6 ± 12.4	253.6 ± 16.7	250.0 ± 17.2	247.2 ± 22.3	0.24

## Data Availability

The data presented in this study are available on request from the corresponding author. The data are not publicly available due to ethical and privacy reasons.
